# Occurrence of butterflies and moths (Insecta, Lepidoptera) in Mordovia State Nature Reserve

**DOI:** 10.3897/BDJ.9.e69813

**Published:** 2021-10-12

**Authors:** Lavr Bolshakov, Alexander Ruchin, Gennady Semishin, Vasiliy Anikin, Vladimir Piskunov, Alexey Matov, Anatoly Semenov, Oleg Artaev

**Affiliations:** 1 Russian Entomological Society, Russian Federation, St. Petersburg, Russia Russian Entomological Society, Russian Federation St. Petersburg Russia; 2 Joint Directorate of the Mordovia State Nature Reserve and National Park "Smolny", Saransk, Russia Joint Directorate of the Mordovia State Nature Reserve and National Park "Smolny" Saransk Russia; 3 Saratov State University, Saratov, Russia Saratov State University Saratov Russia; 4 Vitebsk State University, Vitebsk, Russia Vitebsk State University Vitebsk Russia; 5 Zoological Institute, St Petersburg, Russia Zoological Institute St Petersburg Russia; 6 Papanin Institute for Biology of Inland Waters Russian Academy of Sciences, Borok, Russia Papanin Institute for Biology of Inland Waters Russian Academy of Sciences Borok Russia

**Keywords:** Lepidoptera, biodiversity, protected areas, Central Russia

## Abstract

**Background:**

Faunistic research in protected areas is of greatest interest since these are the most unique places in the region. Many of these are islands of minimal anthropogenic impact, such as the Mordovia State Nature Reserve (Russian Federation), founded in 1936. The purpose of the publication of the basis of faunistic research - occurrences of species, is availability of factual information to a broad range of researchers and its implication in research on a wider scale.

**New information:**

For the first time, a total of 7,606 records of Lepidoptera occurrences from the Mordovia State Nature Reserve with coordinates have been published as a dataset. It is necessary to embed them in the Global Biodiversity Information Facility (GBIF) in order to make them accessible to everyone. As a result of research from 2007 to 2021, more than 600 taxa were identified for the first time for the territory of Mordovia State Nature Reserve, including more than 450 species for the Republic of Mordovia, four species for the Middle Volga Region and eight species for the Middle and Lower Volga Region.

## Introduction

Forests are the most important habitat for numerous invertebrates and vertebrates. Globally, forests are critical to climate, biodiversity and human well-being. Many authors noted the importance of biodiversity in forest ecosystems (Brockerhoff et al. 2017, Ammer et al. 2018, Chifundera 2019). In many parts of the world, forest ecosystems remain untouched and have not been exposed to anthropogenic activities. Such ecosystems are often considered as a biodiversity hotspot and often they are listed as protected areas (Mohd-Azlan et al. 2020, Vieira et al. 2019). Forest protected areas (nature reserves and national parks) can occupy both large and small areas in natural climatic zones and include typical ecosystems of such climatic zones. In the forest natural zone, such areas are untouched woodlands in which the fauna and flora are preserved in their original form (Simonov and Matantseva 2020, Teteryuk et al. 2020, Bondarenko et al. 2020).

Recently, we have published lists of the Lepidoptera fauna of Mordovia State Nature Reserve (Bolshakov et al. 2021a, Bolshakov et al. 2018, Bolshakov et al. 2019, Bolshakov et al. 2021b). However, they did not contain detailed information with coordinates and dates of the occurrence of various Lepidoptera species. The aim of this work is to describe a dataset of up-to-date information on the occurrence of Lepidoptera in the Mordovia State Nature Reserve, which has been recently published in the GBIF as a Darwin Core Archive.

## Sampling methods

### Study extent

The Mordovia State Nature Reserve is located in the Republic of Mordovia (Central Russia). The area covers 321.62 km^2^. It is almost entirely a forest protected area (forests occupy 89.3%). It is mainly covered with pine forests (*Pinussylvestris* Linnaeus). They form pure or mixed plant communities in the southern, central and western parts. Partially, the area is covered by birches (*Betulapendula* Roth). They were formed at the former pine sites which had been cut down in the 1940s and 1950s and burned down in 2010 (Khapugin et al. 2016, Ruchin et al. 2019). In addition, plant communities of other small-leaved tree species (aspen, alder) are formed on burned forest areas. Deciduous forests of *Quercusrobur* Linnaeus and *Tiliacordata* Miller are located mainly in the northern, western and south-western parts. They are common in floodplains. Those forests with dominant species of *Piceaabies* (L.) H. Karsten and *Alnusglutinosa* (L.) Gaertner are also located mainly in the floodplains of small rivers and streams and occupy small areas. Areas of floodplain meadows are situated in the western part of the Mordovia State Nature Reserve. Additionally, there are small areas with meadow vegetation in large clearings previously used as places for outbuildings (Khapugin et al. 2016, Ruchin and Khapugin 2019).

### Sampling description

A variety of collection methods were used for conducting the research: manual collection, light traps and bait traps (Golub et al. 2012). The arrangement of traps tried to diversify the coverage of biotopes and geography of the Nature Reserve. Beer traps were also used for the research. The beer traps are a plastic 5-litre container with a window cut out in it on one side at a distance of 10 cm from the bottom. With the help of a load, a rope with a tied trap was thrown on to a tree branch at a height of 5 to 12 m from the soil surface (Ruchin et al. 2020). As bait, fermenting beer, white and red dry wine were used, with the addition of honey, jam or sugar.

### Quality control

Each observation contains fundamental information, such as location (coordinates), date, name of observer and name of identifier. A large part of the coordinates was determined directly on site with the help of a GPS device. In other cases, these were geolocated with the help of publicly available Soviet topographic maps in scale 1:200,000. The margin of error in the measurement of coordinates was 50 m. The accuracy of determining coordinates was up to the fourth digit. In all cases, the WGS84 coordinate system was used.

Many species were identified by their genitals. Relatively complex groups of Microlepidoptera were primarily determined by the basic domestic manuals (Ler 1999, Ler 1997, Medvedev 1986, Medvedev 1981, Medvedev 1978) and Macrolepidoptera - based on Fibiger 2009, Hausmann 2019 etc. Binoculars MBS-9 and MBS-10 with standard sets of eyepieces and objectives were used.

The material was determined mainly by L. V. Bolshakov and partly by V. I. Piskunov (Vitebsk) and V. V. Anikin (Saratov). Some complex species were identified or tested by S. Yu. Sinev, S. V. Baryshnikova, A. Yu. Matov, A. L. Lvovsky (St. Petersburg), A.V. Sviridov (Moscow) and P. Ya. Ustyuzhanin (Novosibirsk).

## Geographic coverage

### Description

**Figure 1.**
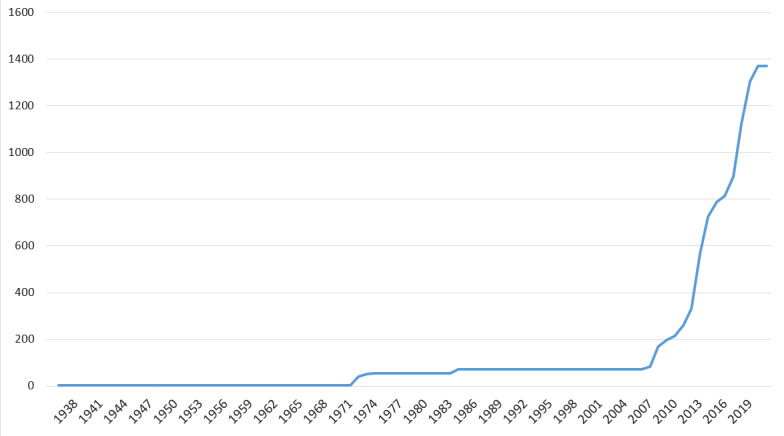
Accumulation curve of the number of butterfly and moth taxa in Mordovia State Nature Reserve (Y-axis) through years (X-axis).

The dataset contains information about the occurrence of Lepidoptera from the territory of Mordovia State Nature Reserve (Russian Federation). The main part of the research was carried out from 2007 - 2021 (Fig. 1). Collection material from previous years was also used.

### Coordinates

54°42'24"N and 54°56'08"N Latitude; 43°37'49"E and 43°04'28"E Longitude.

## Taxonomic coverage

### Description

Most individuals of butterflies and moths were identified to species (7,473) and a small part to genus (133). The taxonomic diversity of the research area is represented by 1372 taxa belonging to 61 families. Given the long-term nature of our research, this is an almost exhaustive list of species that reproduce in the region.

As a result of our research, from 2007 to 2021, more than 600 species were identified for the first time for the Mordovia State Nature Reserve. Previously published works were used as a basis of this research (Antonova 1974, Plavilshchikov 1964, Mozolevskaya et al. 1971, Sviridov and Susarev 2013, Feoktistov 2011). More than 450 species were first identified for the Republic of Mordovia, four species for the Middle Volga Region (*Agrocholamacilenta* (Hübner, 1809), *Tinagmaocnerostomellum* (Stainton, 1850), *Monochroaconspersella* (Herrich-Schäffer, 1854) and*Tiliaceaaurago* (Denis & Schiffermüller, 1775)) and eight species for the Middle and Lower Volga Region (*Monochroasimplicella* (Lienig & Zeller, 1846), *Chionodeselectella* (Zeller, 1839), *Gnorimoschemaherbichii* (Nowicki, 1864), *Caryocolumtricolorella* (Haworth, 1812), *Phaulernisfulviguttella* (Zeller, 1839), *Prochoreutissehestediana* (Fabricius, 1776), *Gynnidomorphavectisana* (Humphreys & Westwood, 1845) and *Lobesiavirulenta* Bae & Komai, 1991). The greatest richness was found in three families: Noctuidae – 233 species, Tortricidae – 210 species, Geometridae – 206 species (Bolshakov et al. 2021).

In this publication, we followed the GBIF taxonomy in most cases, with a few exceptions. In recent publications (Bolshakov et al. 2018, Bolshakov et al. 2019, Bolshakov et al. 2021b, Bolshakov et al. 2021a,Bolshakov and Ismagilov 2020, Kuznetsov and Stekolnikov 2001, Sinev 2019; etc.), Incurvariidae et Prodoxidae were treated as subfamilies within Adelidae s.l. due to the monotonous structure of the genitalia in that group. The Acrolepiidae were interpreted as a family due to the specific morphology of the species (Kuznetsov and Stekolnikov 2001). Phycitidae et Pyraustidae were also considered as families in connection with the peculiarities of morphology (Kuznetsov and Stekolnikov 2001) of the species and ambiguous results of preliminary molecular studies (Regier et al. 2012). The Satyiridae were interpreted as a family due to the peculiarities of morphology (Kuznetsov and Stekolnikov 2001) of species and the results of molecular studies (Wahlberg et al. 2003). Affiliation of Epiplemidae to the Uraniidae was rejected due to differences in morphology of the species (Kuznetsov and Stekolnikov 2001). The Thyatiridae were considered a family due to peculiarities of morphology of the species (Kuznetsov and Stekolnikov 2001). We consider Lemoniidae as a family due to the huge morphological differences from the representatives of Brahmeidae s. str. (Kuznetsov and Stekolnikov 2001).

## Temporal coverage

**Formation period:** 1936; 1948; 1970; 1972; 1973; 2007-2021.

## Usage licence

### Usage licence

Other

### IP rights notes

Creative Commons Attribution (CC-BY) 4.0 License

## Data resources

### Data package title

Occurrence of butterflies and moths (Insecta, Lepidoptera) in Mordovia State Nature Reserve

### Resource link

https://www.gbif.org/dataset/bf77f311-65ad-4008-bce9-0805a0f087d4

### Alternative identifiers

https://doi.org/10.15468/tg288g

### Number of data sets

1

### Data set 1.

#### Data set name

Occurrence of butterflies and moths (Insecta, Lepidoptera) in Mordovia State Nature Reserve

#### Data format

Darwin Core Archive

#### Number of columns

18

#### Character set

UTF-8

#### Data format version

1.2

#### Description

This dataset contains data on the occurrences of Lepidoptera from the territory of Mordovia State Nature Reserve. The dataset contains information on 7,606 occurrences of 1372 taxa from 61 families (Table 1).

**Data set 1.** 

Column label	Column description
occurrenceID	The globally unique identifier number for the record
basisOfRecord	The specific nature of the data record: HumanObservation
eventDate	Date format as YYYY-MM-DD
scientificName	The full scientific name including the genus name and the lowest level of taxonomic rank with the authority
kingdom	The full scientific name of the kingdom in which the taxon is classified
decimalLatitude	The geographic latitude of location in decimal degrees
decimalLongitude	The geographic longitude of location in decimal degrees
coordinateUncertaintyInMetres	The horizontal distance (in metres) from the given decimalLatitude and decimalLongitude describing the smallest circle containing the whole of the Location
geodeticDatum	The ellipsoid, geodetic datum or spatial reference system (SRS) upon which the geographic coordinates given in decimalLatitude and decimalLongitude are based
Country	The name of the country or major administrative unit in which the Location occurs
countryCode	The standard code for the country in which the Location occurs
individualCount	The number of individuals represented present at the time of the Occurrence
year	Year the event was recorded
month	The month the event was recorded
day	The integer day of the month on which the Event occurred
recordedBy	A person or group responsible for recording the original Occurrence
identifiedBy	A list of names of people, who assigned the Taxon to the subject
identificationRemarks	Comments or notes about the Identification

## Figures and Tables

**Table 1.** List of families and respective species richness of Lepidoptera found in Mordovia State Nature Reserve, sorted by number of species

Families	Number of species
Noctuidae	233
Tortricidae	210
Geometridae	206
Gelechiidae	88
Crambidae	84
Erebidae	77
Pyralidae	49
Nymphalidae	46
Elachistidae	39
Notodontidae	27
Lycaenidae	25
Coleophoridae	23
Pterophoridae	21
Tineidae	20
Gracillariidae	19
Sphingidae	16
Lasiocampidae	14
Pieridae	13
Drepanidae	12
Hesperiidae	10
Ypsolophidae	10
Oecophoridae	9
Zygaenidae	9
Adelidae	8
Nepticulidae	7
Nolidae	7
Yponomeutidae	7
Eriocraniidae	6
Scythrididae	6
Papilionidae	5
Psychidae	5
Sesiidae	5
Argyresthiidae	4
Cosmopterigidae	4
Hepialidae	4
Momphidae	4
Chimabachidae	3
Cossidae	3
Lypusidae	3
Blastobasidae	2
Choreutidae	2
Douglasiidae	2
Epermeniidae	2
Glyphipterigidae	2
Incurvariidae	2
Micropterigidae	2
Plutellidae	2
Saturniidae	2
Arctiidae	1
Batrachedridae	1
Brahmaeidae	1
Bucculatricidae	1
Endromidae	1
Limacodidae	1
Lyonetiidae	1
Opostegidae	1
Prodoxidae	1
Roeslerstammiidae	1
Schreckensteiniidae	1
Tischeriidae	1
Uraniidae	1
Total	1372
